# Supplementary material for: Paired assistance and poverty alleviation: Experience and evidence from China
Source: PLoS One. 2024 Feb 16;19(2):e0297173. doi: 10.1371/journal.pone.0297173 (PMC10871504; doi:10.1371/journal.pone.0297173)
Supplement: Attachment — Submitted filename: Revision Comments (PLOS1).docx [file pone.0297173.s002.docx]

Revision Comments

The topic is interesting but a serious level of hard work is required for acceptance and publication in PLOS-ONE.

General Comments:

It is very much astonishing for me that manuscript does not have page numbers and continuous line numbers even. How can I make a review and mention appropriate changes in the various sections of the manuscript. Note that the formatting of the manuscript highly matters for evaluation.

1. What is the background of conducting this study mention in the abstract.
2. Why the study is conducted on Anhui province of China. Is it appropriate to evaluate an entire country situation based on single province data? Justify
3. Mention practical and social implication of the study in abstract.
4. The gap of study is not clear. Mention appropriate reference of the prior literature that why that study is necessary?
5. The research questions are missing in the manuscript.
6. Elaborate the problem of your study using some graphical representations in introduction.
7. Poverty is a global issue but what is the contribution of China in reducing poverty compare with world? Draw some graphs related to this in introduction section.
8. Separate the literature review section from introduction.

Enrich the literature review by the below mentioned studies

<https://link.springer.com/article/10.1007/s11356-021-17099-w>

<http://ramss.spcrd.org/index.php/ramss/article/view/255>

9. Give the summary of literature review with appropriate research gap.
10. Separate the sections of literature review according to the linkage of variables.
11. Provide a related theocratical support for your study.
12. Methodology section missing the information and sources of research variables. Provide a table for the sources, measuring units, and descriptions of the variables.
13. Where, is the conceptual framework of the study? Where, are the research hypothesis? Provide a diagram that shows the connections of variables.
14. Give the full form of PSM-DID method.
15. You have used both primary and secondary data give logical reasons?
16. What do you think the regressed data span is sufficient for some empirical analysis?
17. Check the third mathematical equation. Also, verify all the mathematical equations in the manuscript.
18. The conclusion section needs more explanation and practical policy implications section after the conclusion.
