# Supplementary material for: Paired assistance and poverty alleviation: Experience and evidence from China
Source: PLoS One. 2024 Feb 16;19(2):e0297173. doi: 10.1371/journal.pone.0297173 (PMC10871504; doi:10.1371/journal.pone.0297173)
Supplement: Attachment — Submitted filename: Response to Reviewers.docx [file pone.0297173.s003.docx]

**Reviewer #1: Authors of “Paired assistance and poverty alleviation: Experience and evidence from China” have written an interesting paper and put a lot of effort and can be accepted after major corrections. I have the following suggestions.**

**The current scope of the literature review is restricted in its coverage. To enhance its comprehensiveness, authors should incorporate recent advancements from the field.**

**In the introduction, it is imperative to articulate the research questions that will guide this study.**

**Within the literature review section, a distinct and unequivocal hypothesis should be put forth to provide a clear direction for the research.**

**To improve the overall readability of the paper, it is advised to reduce the excessive use of first-person pronouns.**

**A comprehensive discussion of the results is needed, along with an exploration of how these findings contribute to the development of the underlying theoretical framework.**

**In general, it is recommended to revise the reference list to encompass the latest advancements in the literature concerning post-COVID-19 poverty alleviation. Furthermore, it is pertinent to outline potential future directions pertaining to sustainable development. To augment the reference list, the following pertinent literature should be included:**

**https://doi.org/10.1108/IJOEM-11-2020-1417
https://doi.org/10.1142/S021964922240010X
https://doi.org/10.1016/j.jbef.2020.100341
https://doi.org/10.1016/j.frl.2020.101640
https://doi.org/10.1007/s11356-023-26631-z
https://doi.org/10.1007/s11356-022-24458-8
https://doi.org/10.1016/j.enpol.2011.02.026
https://doi.org/10.1016/j.jclepro.2020.125143**

**Answer #1**

Thanks to the suggestions of review experts, the introduction has been revised and corresponding literature has been added. The research hypothesis is put forward in the third part of the Conceptual Framework. At the same time, the use of the first person is reduced, and the conclusion is modified according to the suggestions of experts

**Reviewer #2: Revision Comments**

Thanks for the suggestions of the reviewers, and the following reply is made in response to the opinions of the experts. As for the format problem, I am sorry that I did not add the page number and line number, but it has been added in the revised version. In response to the expert opinions, I have made each reply below

1. **What is the background of conducting this study mention in the abstract.**

The background of conducting this study, as mentioned in the abstract, is based on China's significant success in poverty alleviation over the past 40 years. The country has lifted nearly 800 million people out of poverty, representing more than 75% of the global population reduction in poverty during the same period. This extraordinary achievement suggests that China's experience could be informative for other countries addressing poverty.
**2. Why the study is conducted on Anhui province of China. Is it appropriate to evaluate an entire country situation based on single province data? Justify**

Anhui province, located in the eastern region of China, has a mixed economy with agricultural and industrial sectors. It has also been a focus of poverty alleviation efforts. Therefore, the findings from Anhui province could possibly provide valuable insights that are representative of other similar regions in China. In conclusion, while the findings from a single province study should not be directly extrapolated to the entire country, they contribute valuable knowledge to the broader understanding of the issue and can inform and guide further research and policy-making.
**3. Mention practical and social implication of the study in abstract.。**

Our findings reveal that both governmental and social assistance play significant roles in poverty alleviation, with their relative effectiveness dependent on specific conditions and contexts. This has important practical implications for policy-making, suggesting that an effective poverty alleviation strategy should involve a combination of governmental and social efforts tailored to the specific needs and circumstances of the targeted poor households. Furthermore, our study has significant social implications. By improving the effectiveness of poverty alleviation strategies, we can help alleviate the hardships faced by poor households and contribute to social equity and stability. Additionally, understanding the role of social forces in poverty alleviation can facilitate better collaboration between government entities, non-governmental organizations, and communities, fostering a collective approach to tackling poverty.
**4. The gap of study is not clear. Mention appropriate reference of the prior literature that why that study is necessary?**

This research is necessary as it can inform more effective and targeted poverty alleviation policies in China, a country with a significant rural poor population. Furthermore, our findings can potentially offer insights for other developing contexts with similar socio-economic conditions. Our study contributes to the ongoing discourse on poverty alleviation strategies and their implementation, and provides valuable empirical evidence for policy-makers, researchers, and practitioners in the field.
**5. The research questions are missing in the manuscript.**

Thanks to the suggestions of the reviewers, changes have been made in the introduction.
**6. Elaborate the problem of your study using some graphical representations in introduction.**

Thanks to the suggestions of the reviewers, it has been added to the introduction.
**7. Poverty is a global issue but what is the contribution of China in reducing poverty compare with world? Draw some graphs related to this in introduction section.**

Thanks to the suggestions of the reviewers, it has been added to the introduction
**8. Separate the** **literature review section from introduction. Enrich the literature review by the below mentioned studies.
https://link.springer.com/article/10.1007/s11356-021-17099-w
http://ramss.spcrd.org/index.php/ramss/article/view/255**

Thanks to the suggestions of reviewers, the literature review and introduction have been separated, and the literature review is the second part and the corresponding literature is added
**9. Give the summary of literature review with appropriate research gap.**

Thanks to the suggestions of the reviewers, the literature review has been summarized
**10. Separate the sections of literature review according to the linkage of variables.**

Thanks to the suggestions of the reviewers , the logic of the literature review in this paper starts from China's poverty alleviation policies, emphasizing that China's policies mobilize social forces to participate in poverty alleviation → to existing studies that do not pay enough attention to the mechanism and effect of social forces → Finally, it points out that there are still gaps in the research on social forces for poverty alleviation that need further attention.
**11. Provide a related theocratical support for your study.**

Thanks to the suggestions of the reviewers, a section of theoretical support was added to the first section of the literature review
**12. Methodology section missing the information and sources of research variables. Provide a table for the sources, measuring units, and descriptions of the variables.**

Thanks for the comments of reviewers, the variables in this paper mainly come from field survey data, the specific sources are mentioned in the data sources, and the measurement and explanation are mentioned in the Methods section
**13. Where, is the conceptual framework of the study? Where, are the research hypothesis? Provide a diagram that shows the connections of variables.**

Thanks to the opinions of review experts, the Conceptual Framework and research hypothesis are put in the third part of the Conceptual Framework, which is described in words. The research assumptions presented in 3.2 Empirical expectations have been revised to make them more visible and easy to view.
**14.Give the full form of PSM-DID method.**

Thanks to the opinions of review experts, PSM-DID is a combination of two methods. In this paper, PSM is done first and then DID. The two forms are separate
**15.You have used both primary and secondary data give logical reasons?**

Thanks to the opinions of review experts. Both primary and secondary data have been employed, reflecting a methodological progression in our research approach. The two factors present a progressive relationship.
**16. What do you think the regressed data span is sufficient for some empirical analysis?**

As the author of this study, I believe that our data span from 2016 to 2019 is sufficient for our empirical analysis. Here are my reasons:

Research Objectives: Our study aims to analyze the effects of paired assistance on poverty alleviation, particularly comparing the effects of governmental assistance and social assistance. The four-year span provides an adequate period to observe the impacts of these assistance strategies.

Data Quality: The data we used is of high quality, detailing the conditions of 2031 registered poor households and tracking them continuously throughout the study period. This provides us with a robust basis for understanding the effects of poverty alleviation strategies.

Contextual Relevance: Our study is specifically focused on recent poverty alleviation efforts in Anhui Province. The chosen data span aligns with the timeline of these efforts, ensuring the relevance and applicability of our findings.

While we acknowledge that longer data spans could provide additional insights, especially for understanding long-term trends, we believe that our current data span is suitable for the scope and objectives of our study.
**17. Check the third mathematical equation. Also, verify all the mathematical equations in the manuscript.**

We appreciate the insightful advice from the reviewers. Modifications to the equation have been made and thoroughly reviewed.
**18. The conclusion section needs more explanation and practical policy implications section after the conclusion.**

Thanks to the opinions of review experts, the conclusion is modified according to the suggestions of experts.
